# Supplementary material for: B-Cell Epitope Mapping of the Vibrio cholera Toxins A, B, and P and an ELISA Assay
Source: Int J Mol Sci. 2022 Dec 28;24(1):531. doi: 10.3390/ijms24010531 (PMC9820764; doi:10.3390/ijms24010531)
Supplement: Supplementary file 1 [file ijms-24-00531-s001.zip › ijms-2011001-supplementary.pdf]

# B Cell Epitope Mapping of the *Vibrio cholera* Toxins A, B, and P and an ELISA assay

**Salvatore G. De-Simone [1,2,3,*], Paloma Napoleão-Pêgo [1,2], Priscilla S. Gonçalves [1,2,3], Guilherme C. Lechuga [1,2], Sergian V. Cardoso [4], David W. Provance, Jr. [1,2], Carlos M. Morel [1] and Flavio R. da Silva [1,2]**

1Center for Technological Development in Health (CDTS)/National Institute of Science and Technology for Innovation in Neglected Diseases Populations (INCT-IDPN), Oswaldo Cruz Foundation (FIOCRUZ), Rio de Janeiro 21040-900, RJ, Brazil

2Epidemiology and Molecular Systematics Laboratory (LEMS), Oswaldo Cruz Institute, Oswaldo Cruz Foundation (FIOCRUZ), Rio de Janeiro 21040-900, RJ, Brazil

3Program of Post-Graduation on Science and Biotechnology, Molecular and Cellular Biology Department, Biology Institute, Federal Fluminense University, Niterói 24020-036, RJ, Brazil

4Department of Health, Graduate Program in Translational Biomedicine (BIOTRANS), University of Grande Rio (UNIGRANRIO), Caxias 25071-202, RJ, Brazil

* Correspondence: dsimone@cdts.fiocruz.br; Tel.: +55-21-3865-8182

**Supplementary Figures**

Figure S1- List of Vibrio cholerae [toxin A (P01555), B (P01556), and P (P29485)] synthetic peptides and position in the cellulose membrane of Spot synthesis.

Figure S2- Purification of the peptide B (Vc/TxB-11) by HPLC using an XBridge BEH C18 (2.7 μ, 5 cm x 4.6 mm) column coupled to a Water HPLC system at a flow rate of 1.2 ml $min^{-1}$ using mobile phases A [0.05% formic acid in water (18 MΩ × cm)] and B [(0.05% formic acid in ACN (acetonitrile acid)] (v/v) in water. Detection at 200-300 nm using a diode array.

Figure S3- Mass spectrometry. The peptide B (Vc/TxB-11) was solubilized in deionized water to a final concentration of 10 μg/ml and then added formic acid to a final concentration of 0.1%. The mass spectrometer used was the Water UPLC model Acquity-I Class. The samples were electronically injected by the equipment at 1 μl/min. The range used for ion detection ranged from 1000-11500 m/z.

| Peptide | Sequence | Peptide | Sequence | Peptide | Sequence |
|---|---|---|---|---|---|
| A1 | GSGSGMVKIIFVFFI | B1 | NDVLGAYSPHPDEQE | C1 | VKRQIFSGYQSDIDT |
| A2 | MVKIIFVFFIFLSSF | B2 | AYSPHPDEQEVSALG | C2 | FSGYQSDIDTHNRIK |
| A3 | FVFFIFLSSFSYAND | B3 | PDEQEVSALGGIPYS | C3 | SDIDTHNRIKDELGS |
| A4 | FLSSFSYANDDKLYR | B4 | VSALGGIPYSQIYGW | C4 | DTHNRIKDELGSGSG |
| A5 | SYANDDKLYRADSRP | B5 | GIPYSQIYGWYRVHF | C5 | GSGSGMIKLKFGVFF |
| A6 | DKLYRADSRPPDEIK | B6 | QIYGWYRVHFGVLDE | C6 | MIKLKFGVFFTVLLS |
| A7 | ADSRPPDEIKQSGGL | B7 | YRVHFGVLDEQLHRN | C7 | FGVFFTVLLSSAYAH |
| A8 | PDEIKQSGGLMPRGQ | B8 | GVLDEQLHRNRGYRD | C8 | TVLLSSAYAHGTPQN |
| A9 | QSGGLMPRGQSEYFD | B9 | QLHRNRGYRDRYYSN | C9 | SAYAHGTPQNITDLC |
| A10 | MPRGQSEYFDRGTQM | B10 | RGYRDRYYSNLDIAP | C10 | GTPQNITDLCAEYHN |
| A11 | SEYFDRGTQMNINLY | B11 | RYYSNLDIAPAADGY | C11 | ITDLCAEYHNTQIYT |
| A12 | RGTQMNINLYDHARG | B12 | LDIAPAADGYGLAGF | C12 | AEYHNTQIYTLNDKI |
| A13 | NINLYDHARGTQTGF | B13 | AADGYGLAGFPPEHR | C13 | TQIYTLNDKIFSYTE |
| A14 | DHARGTQTGFVRHDD | B14 | GLAGFPPEHRAWREE | C14 | LNDKIFSYTESLAGK |
| A15 | TQTGFVRHDDGYVST | B15 | PPEHRAWREEPWIHH | C15 | FSYTESLAGKREMAI |
| A16 | VRHDDGYVSTSISLR | B16 | AWREEPWIHHAPPGC | C16 | SLAGKREMAIITFKN |
| A17 | GYVSTSISLRSAHLV | B17 | PWIHHAPPGCGNAPR | C17 | REMAIITFKNGAIFQ |
| A18 | SISLRSAHLVGQTIL | B18 | APPGCGNAPRSSMSN | C18 | ITFKNGAIFQVEVPG |
| A19 | SAHLVGQTILSGHST | B19 | GNAPRSSMSNTCDEK | C19 | GAIFQVEVPGSQHID |
| A20 | GQTILSGHSTYYIYV | B20 | SSMSNTCDEKTQSLG | C20 | VEVPGSQHIDSQKKA |
| A21 | SGHSTYYIYVIATAP | B21 | TCDEKTQSLGVKFLD | C21 | SQHIDSQKKAIERMK |
| A22 | YYIYVIATAPNMFNV | B22 | TQSLGVKFLDEYQSK | C22 | SQKKAIERMKDTLRI |
| A23 | IATAPNMFNVNDVLG | B23 | VKFLDEYQSKVKRQI | C23 | IERMKDTLRIAYLTE |
| A24 | NMFNVNDVLGAYSPH | B24 | EYQSKVKRQIFSGYQ | C24 | DTLRIAYLTEAKVEK |
| | | | | | |
| D1 | AYLTEAKVEKLCVWN | E1 | DEHKTLIENVKLQGY | F1 | YEQKTLECTKNGSGS |
| D2 | AKVEKLCVWNNKTPH | E2 | LIENVKLQGYRINII | F2 | EQKTLECTKNGSGSG |
| D3 | LCVWNNKTPHAIAAI | E3 | KLQGYRINIIQVIVS | F3 | |
| D4 | NKTPHAIAAISMANG | E4 | RINIIQVIVSENVVD | F4 | |
| D5 | HAIAAISMANGSGSG | E5 | QVIVSENVVDEADCS | F5 | |
| D6 | GSGSGMGYVRVIYQF | E6 | ENVVDEADCSQKKSV | F6 | |
| D7 | MGYVRVIYQFPDNLW | E7 | EADCSQKKSVKERIK | F7 | |
| D8 | VIYQFPDNLWWNECT | E8 | QKKSVKERIKIEWGK | F8 | |
| D9 | PDNLWWNECTNQVYY | E9 | KERIKIEWGKINVVP | F9 | |
| D10 | WNECTNQVYYAQDPM | E10 | IEWGKINVVPYLVFS | F10 | |
| D11 | NQVYYAQDPMKPERL | E11 | INVVPYLVFSALYVA | F11 | |
| D12 | AQDPMKPERLIGTPS | E12 | YLVFSALYVALLPVI | F12 | |
| D13 | KPERLIGTPSIIQTK | E13 | ALYVALLPVIWWSYG | F13 | |
| D14 | IGTPSIIQTKLLKIL | E14 | LLPVIWWSYGQWYQH | F14 | |
| D15 | IIQTKLLKILCEYHP | E15 | WWSYGQWYQHELAGI | F15 | |
| D16 | LLKILCEYHPAPCPN | E16 | QWYQHELAGITHDLR | F16 | |
| D17 | CEYHPAPCPNDQIIK | E17 | ELAGITHDLRDLARL | F17 | |
| D18 | APCPNDQIIKALWPH | E18 | THDLRDLARLPGITI | F18 | |
| D19 | DQIIKALWPHGFISS | E19 | DLARLPGITIQKLSE | F19 | |
| D20 | ALWPHGFISSESLTQ | E20 | PGITIQKLSEQKLTF | F20 | |
| D21 | GFISSESLTQAIKRT | E21 | QKLSEQKLTFAIDQH | F21 | |
| D22 | ESLTQAIKRTRDFLN | E22 | QKLTFAIDQHQCSVN | F22 | |
| D23 | AIKRTRDFLNDEHKT | E23 | AIDQHQCSVNYEQKT | F23 | |
| D24 | RDFLNDEHKTLIENV | E24 | QCSVNYEQKTLECTK | F24 | |
| | | | | | |
| G1 | | G9 | QEVRKYFCV | G17 | QEVRKYFCV |
| G2 | | G10 | | G18 | |
| G3 | KEVPALTAVETGATN | G11 | KEVPALTAVETGATN | G19 | |
| G4 | | G12 | | G20 | |
| G5 | GYPKDGNAFNNLDRI | G13 | GYPKDGNAFNNLDRI | G21 | |
| G6 | | G14 | | G22 | |
| G7 | YDYDVPDYAGYPYDV | G15 | YDYDVPDYAGYPYDV | G23 | |
| G8 | | G16 | | G24 | YDYDVPDYAGYPYDV |

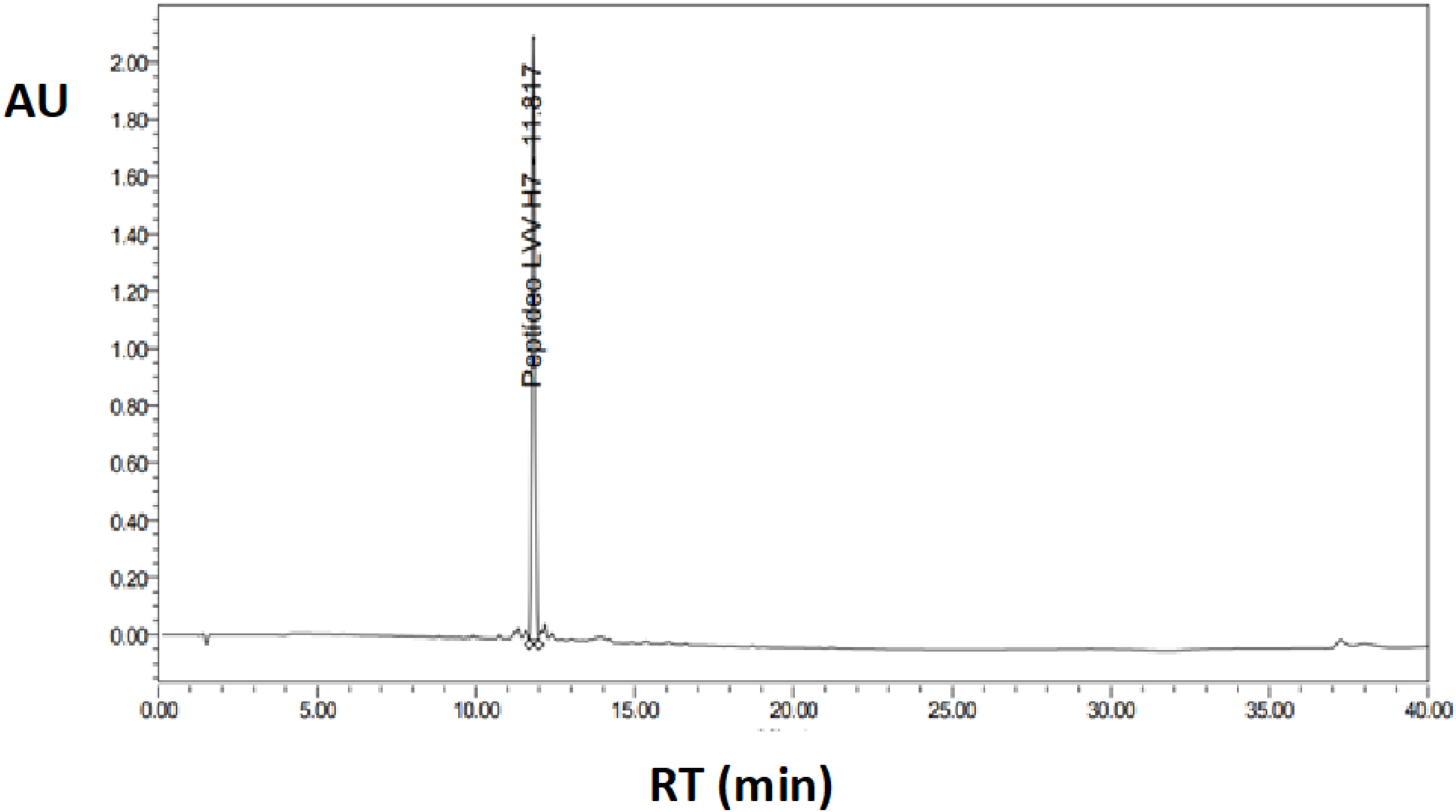

AU
2.00
1.80
1.60
1.40
1.20
1.00
0.80
0.60
0.40
0.20
0.00
Peptideo LVV-H7 - 11.817
0.00
5.00
10.00
15.00
20.00
25.00
30.00
35.00
40.00
RT (min)

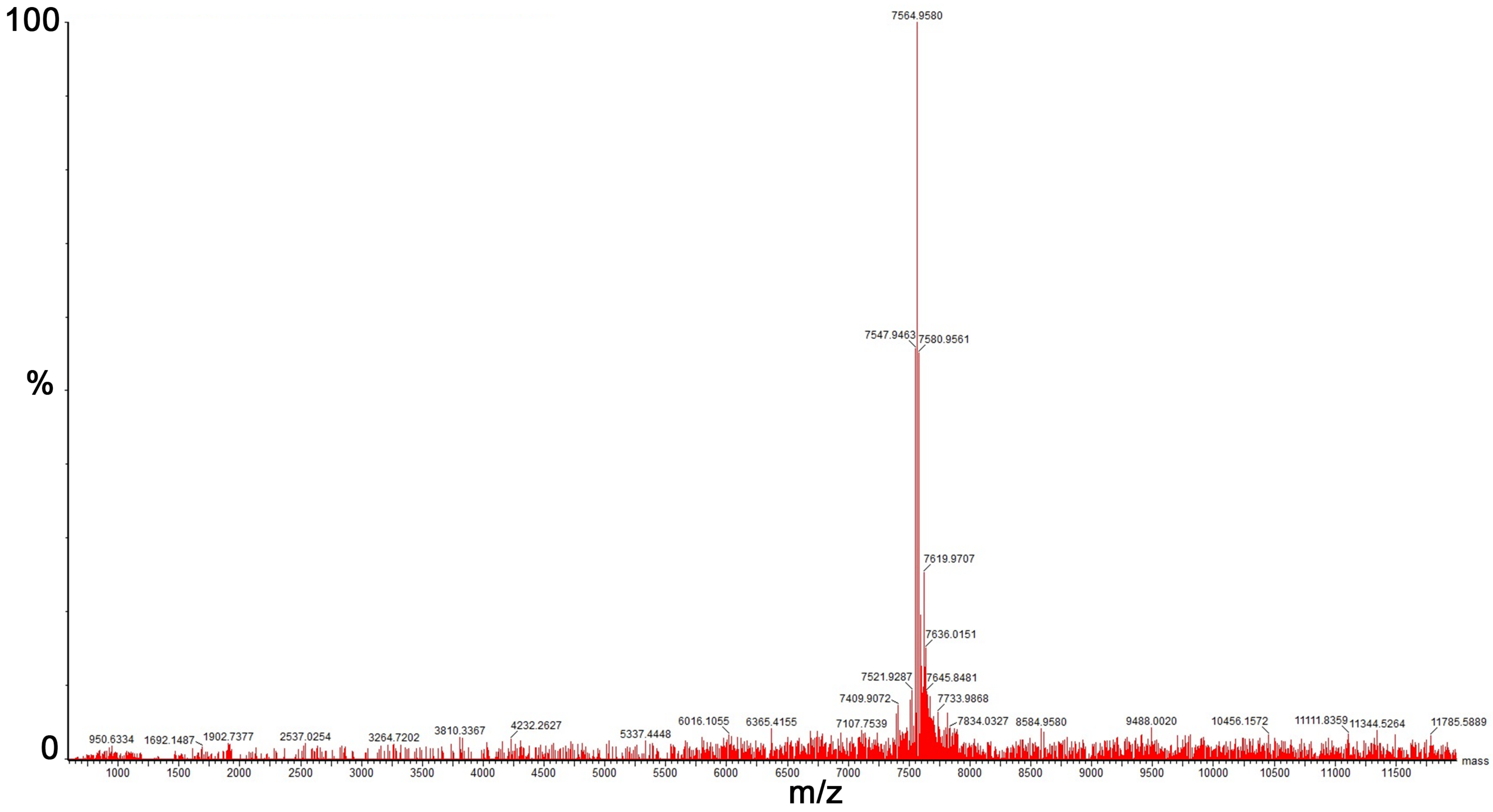
100
%
0
7564.9580
7547.9463
7580.9561
7619.9707
7636.0151
7521.9287
7645.8481
7409.9072
7733.9868
7834.0327
950.6334
1692.1487
1902.7377
2537.0254
3264.7202
3810.3367
4232.2627
5337.4448
6016.1055
6365.4155
7107.7539
8584.9580
9488.0020
10456.1572
11111.8359
11344.5264
11785.5889
1000
1500
2000
2500
3000
3500
4000
4500
5000
5500
6000
6500
7000
7500
8000
8500
9000
9500
10000
10500
11000
11500
mass
m/z
